# Metformin Reduces NGF-Induced Tumour Promoter Effects in Epithelial Ovarian Cancer Cells

**DOI:** 10.3390/ph13100315

**Published:** 2020-10-16

**Authors:** Maritza P. Garrido, Renato Salvatierra, Manuel Valenzuela-Valderrama, Christopher Vallejos, Nicole Bruneau, Andrea Hernández, Margarita Vega, Alberto Selman, Andrew F. G. Quest, Carmen Romero

**Affiliations:** 1 Laboratorio de Endocrinología y Biología de la Reproducción, Hospital Clínico Universidad de Chile, Santiago 8380456, Chile; mgarrido@hcuch.cl (M.P.G.); rsalvatierratm@gmail.com (R.S.); chris.vallejosmoraga@gmail.com (C.V.); nicole.bruneau@ug.uchile.cl (N.B.); ahernandezj@hcuch.cl (A.H.); mvega@hcuch.cl (M.V.); 2 Departamento de Obstetricia y Ginecología, Facultad de Medicina, Universidad de Chile, Santiago 8380453, Chile; selmanalberto@gmail.com; 3 Laboratorio de Microbiología Celular, Instituto de Investigación e Innovación en Salud, Facultad de Ciencias de la Salud, Universidad Central de Chile, Santiago 8320000, Chile; manuel.valenzuela@ucentral.cl; 4 Instituto Nacional del Cáncer, Santiago 8380455, Chile; 5 Laboratorio de Comunicaciones Celulares, Centro de estudios en Ejercicio, Metabolismo y Cáncer (CEMC), Programa de Biología Celular y Molecular, Instituto de Ciencias Biomédicas (ICBM), Facultad De Medicina, Universidad de Chile, Santiago 8380453, Chile; 6 Advanced Center for Chronic Diseases (ACCDiS), Santiago 8380000, Chile

**Keywords:** metformin, epithelial ovarian cancer, NGF, VEGF, survivin, c-MYC, β-catenin

## Abstract

Epithelial ovarian cancer (EOC) is a lethal gynaecological neoplasm characterized by rapid growth and angiogenesis. Nerve growth factor (NGF) and its high affinity receptor tropomyosin receptor kinase A (TRKA) contribute to EOC progression by increasing the expression of c-MYC, survivin and vascular endothelial growth factor (VEGF) along with a decrease in microRNAs (miR) 23b and 145. We previously reported that metformin prevents NGF-induced proliferation and angiogenic potential of EOC cells. In this study, we sought to obtain a better understanding of the mechanism(s) by which metformin blocks these NGF-induced effects in EOC cells. Human ovarian surface epithelial (HOSE) and EOC (A2780/SKOV3) cells were stimulated with NGF and/or metformin to assess the expression of c-MYC, β-catenin, survivin and VEGF and the abundance of the tumor suppressor miRs 23b and 145. Metformin decreased the NGF-induced transcriptional activity of MYC and β-catenin/T-cell factor/lymphoid enhancer-binding factor (TCF-Lef), as well as the expression of c-MYC, survivin and VEGF in EOC cells, while it increased miR-23b and miR-145 levels. The preliminary analysis of ovarian biopsies from women users or non-users of metformin was consistent with these in vitro results. Our observations shed light on the mechanisms by which metformin may suppress tumour growth in EOC and suggest that metformin should be considered as a possible complementary therapy in EOC treatment.

## 1. Introduction

Epithelial ovarian cancer (EOC), the most common subtype of ovarian cancer, is one of the most lethal gynaecological neoplasms in women worldwide, and is characterized by non-specific symptoms and late diagnosis, which result in poor survival rates [1,2,3]. Unfortunately, the current therapies yield modest results. Therefore, it is important to understand the molecular mechanisms that characterize this disease and to develop new strategies to improve patient therapy and survival. EOC is characterized by rapid growth and pronounced angiogenesis, which is attributed to overexpression of many growth factors that favour tumorigenesis, including nerve growth factor (NGF) [4] and vascular endothelial growth factor (VEGF) [5,6].

Some studies have shown that presence and expression of NGF and its high affinity receptor TRKA increase during EOC progression, and promote tumour cell proliferation and angiogenesis by activation of the phosphoinositide 3-kinases (PI3K)/ Protein kinase B (AKT) and mitogen-activated protein kinases (MAPK)/extracellular signal-regulated kinase (ERK) signalling pathways [4,7,8,9]. Moreover, results from our group showed that NGF/TRKA is highly expressed in EOC cells compared to non-tumoral ovarian cells, which leads to increased NGF/TRKA signalling [4]. In addition, NGF stimulation increases the expression of VEGF and the transcription factor c-MYC in EOC cells and explants from EOC biopsies [8,10]. NGF/TRKA signalling (via PI3K/AKT and MAPK/ERK pathways) increases the expression of oncoproteins such as survivin and β-catenin, in cancer cells [11,12,13,14,15], and these two proteins are also upregulated in EOC [16,17].

Survivin is a protein that inhibits apoptosis, regulates cell division and is involved in tissue healing after injury [18,19]. Survivin belongs to the inhibitor of apoptosis (IAP) anti-apoptotic family of proteins and its aberrant expression is associated with increased tumour proliferation, progression, angiogenesis, resistance to therapy, and poor prognosis in several cancers [20,21,22,23]. In addition, survivin expression is a marker of poor prognosis in ovarian cancer patients [24] and its expression promotes VEGF-induced tumour angiogenesis via PI3K/AKT [25]. On the other hand, β-catenin is a transcriptional co-regulator and an adaptor protein for intracellular adhesion [26]. Binding of β-catenin to the T-cell factor/lymphoid enhancer-binding factor (TCF/Lef) family of transcription factors promotes transcription of many oncogenes following activation of the canonical Wnt signalling pathway [27,28,29]. For instance, the β-catenin/TCF-Lef complex increases the expression of proteins associated with epithelial-mesenchymal transition (EMT) [30,31], as well as those relating to cell survival and cell proliferation, including survivin and c-MYC [32,33]. Both proteins in turn promote β-catenin/TCF-Lef activity, in an amplification loop that could be involved in tumour growth and EOC progression.

Because NGF/TRKA signalling regulates the expression of several oncoproteins, we propose that this may involve control via microRNAs (miRs), the largest family of non-coding RNAs [34] that target specific messenger RNAs (mRNAs) to either induce their degradation or block protein translation [34]. The miR-145 and miR-23b are two oncosuppressor miRs that are known to be downregulated in ovarian cancer tissues [35] and in-silico analysis revealed that c-MYC and VEGF could be targets for these miRs (Table 1), which in turn are modulated by NGF/TRKA [35].

Among the different drugs that can block the effects of growth factors in cancer cells, metformin has emerged as an interesting candidate. Metformin is a biguanide, which is widely used to treat metabolic disorders, such as type II Diabetes Mellitus, gestational Diabetes, and Metabolic Syndrome [36,37,38]. Interestingly, metformin has been attributed anti-tumour effects because it decreases the mortality due to cancer in diabetic patients [39]. Furthermore, observational studies in EOC have shown that metformin intake is associated with a decrease in ovarian cancer incidence and mortality [40]. This is particularly intriguing given that metformin is cheap, readily accessible and safe. Mechanisms proposed to explain metformin’s pleiotropic effects include the down-regulation of several oncogenic proteins in different cancer cells, both by epigenetic [41,42], as well as post-transcriptional mechanisms that include miR-mediated regulation [43,44,45].

We previously reported that the anti-diabetic drug metformin reduced NGF-induced proliferation of EOC cells, as well as the angiogenic potential of endothelial cells [46]. So, the purpose of this study was to understand better the mechanisms by which metformin blocks NGF-dependent effects in EOC cells and thereby contributes to clarifying the anti-tumour mechanisms of this drug.

Our results show that metformin, strongly decreases c-MYC, β-catenin and VEGF expression in EOC cells with little effect on non-tumour ovarian cells. Importantly, metformin blocks the NGF-induced increase in c-MYC, survivin and VEGF, as well as the increase in MYC and β-catenin/TCF-Lef transcriptional activity in ovarian cancer cells. Additionally, metformin treatment of EOC cells increased the levels of miR-145 and miR-23b by blocking the NGF-induced decrease in these miRs, suggesting that the anti-tumour effects of metformin could be mediated by miR regulation. Consistent with the in-vitro results, the comparison within a small group of patients with borderline ovarian tumours of users vs. non-users of metformin revealed that metformin intake correlated with decreased immunodetection of survivin, c-MYC and β-catenin in tissue sections. Thus, the results obtained by analysis of human tissue samples confirmed our observations in EOC cell lines.

In summary, these results contribute to a better understanding of the tumour suppressor effects of metformin, by showing that the drug decreases the expression of several important oncoproteins, including c-MYC, VEGF and β-catenin, and likely does so via a pathway involving miR modulation. Thus, metformin holds considerable promise as a possible complementary therapy in EOC treatment.

## 2. Results

### 2.1. Metformin Blocks the NGF-Induced Increase in c-MYC Protein Levels and Transcriptional Activity in Ovarian Cancer Cells

**Figure 1:**
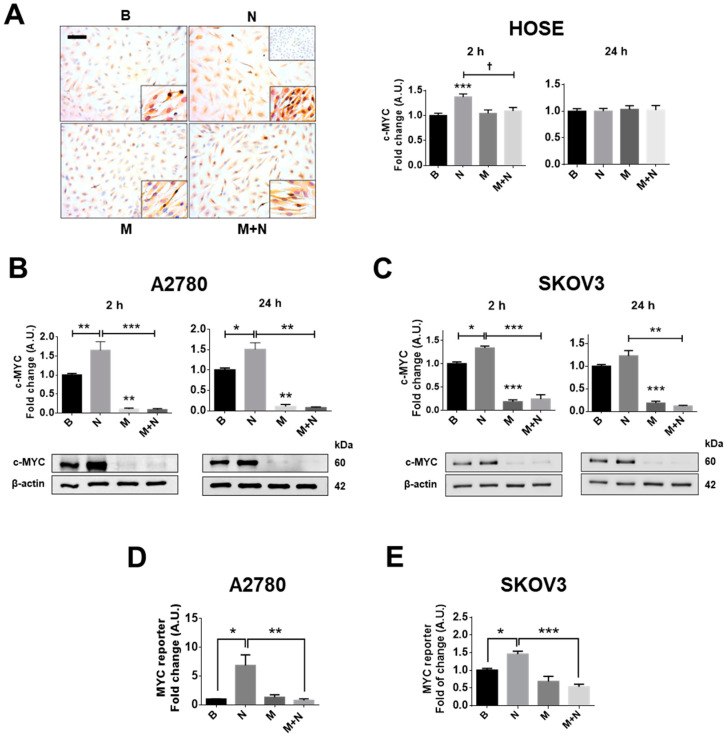
Metformin blocks the nerve growth factor (NGF)-mediated effects on c-MYC in ovarian cells. Ovarian cells were treated with metformin 10 mM for 48 h and/or NGF 100 ng/mL or 150 ng/mL (A2780/human ovarian surface epithelial HOSE cells and SKOV3 cells, respectively) for 24 h or the last 2 h. (**A**) Representative Images of c-MYC immunodetection in HOSE cells with semi-quantification analysis. Bar = 100 µm. Lower right inserts: 400× magnification. Upper right insert: negative control (cells without primary antibody). *N* = 4 independent experiments (8 images were evaluated per experiment). (**B**,**C**) Western blots of c-MYC in A2780 and SKOV3 cells. (**D**,**E**) Gen-reporter assays to evaluate MYC transcriptional activity in the epithelial ovarian cancer (EOC) cells A2780 and SKOV3. *N* = 4 independent experiments. * *p* < 0.05; ** *p* < 0.01 and *** *p* < 0.001. Statistical analysis: Kruskal–Wallis test and Dunn’s post-test. B: basal condition (without stimuli), N: NGF, M: metformin treatment. Results are expressed as the mean ± standard error of the mean (SEM).

Previous results from our group showed that NGF/TRKA increases the proliferation of EOC cells [46] (see Supplementary Figures S1 and S2) and c-MYC protein levels in EOC explants [7,8]. Thus, we evaluated whether NGF and metformin modulated c-MYC protein levels and transcriptional activity in ovarian cell lines following short or long periods of NGF stimulation (2 or 24 h) which were chosen based on results obtained in previous experiments [4,10,46]. The current results show that NGF (100 ng/mL) increases c-MYC protein levels in human ovarian surface epithelial HOSE, as well as A2780 and SKOV3 EOC cells, mainly following short incubation times (2 h; *p* < 0.01, *p* < 0.01 and *p* < 0.05, respectively; Figure 1A,C, Supplementary Figure S3) Alternatively, metformin incubation (10 mM, 48 h) strongly decreased c-MYC protein levels in the EOC cell lines (*p* < 0.01 and *p* < 0.001: Figure 1B,C), but did not decrease c-MYC protein levels compared with the baseline condition (without stimulation) in the non-tumour cell line HOSE (Figure 1A). Because c-MYC is a transcription factor, we determined the transcriptional activity following NGF and metformin incubation. The results show that NGF increased the transcriptional activity of MYC in ovarian cancer cell lines (*p* < 0.05; Figure 1D,E). As expected, metformin treatment blocked the increase in c-MYC protein levels in all the ovarian cell lines (*p* < 0.05; Figure 1A–C), and prevented the increase in MYC transcriptional activity triggered by NGF (*p* < 0.01; Figure 1D,E).

### 2.2. Metformin Treatment Prevents the Increase in β-Catenin/TCF-Lef Transcriptional Activity Induced by NGF in Ovarian Cancer Cells

**Figure 2:**
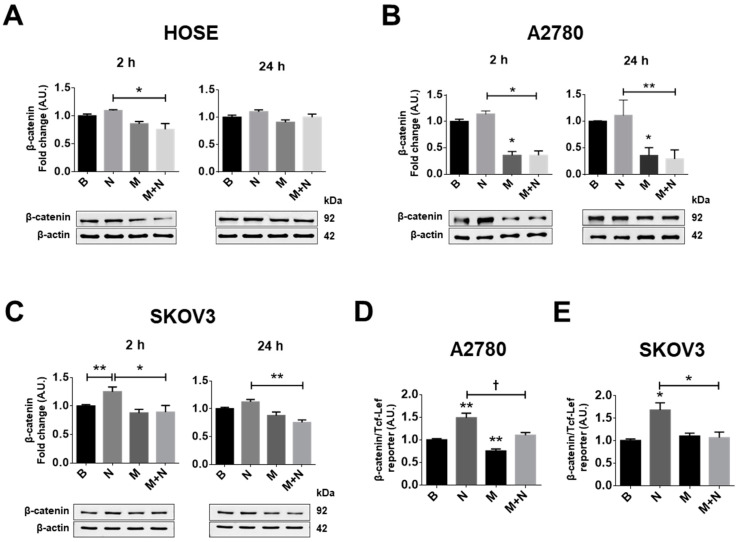
Metformin decreases the NGF-induced β-catenin/TCF-Lef transcriptional activity in EOC cells. Ovarian cells were treated with metformin 10 mM for 48 h and/or NGF 100 ng/mL or 150 ng/mL (A2780/HOSE cells and SKOV3 cells, respectively) for 24 h or the last 2 h. (**A**–**C**) Western blots of β-catenin in HOSE, A2780 and SKOV3 cells after the respective treatments. (**D**,**E**) Gene reporter assays to evaluate β-catenin/TCF-Lef transcriptional activity in the EOC cells A2780 and SKOV3. *N* = 4 independent experiments. * *p* < 0.05 and ** *p* < 0.01. Statistical analysis: Kruskal–Wallis test and Dunn’s post-test. ^†^ *p* < 0.05 as indicated according Mann–Whitney test. B: basal condition (without stimuli), N: NGF, M: metformin treatment. Results are expressed as the mean ± standard error of the mean (SEM).

Because β-catenin is a target protein downstream of AKT signalling [47,48] and NGF activates the AKT pathway (see supplementary Figure S4) we determined whether NGF and metformin modulated the protein levels and the transcriptional activity of β-catenin/TCF-Lef. Under the experimental conditions tested, NGF did not increase the protein levels of β-catenin in HOSE or A2780 cells (Figure 2A,B), but did in SKOV3 cells when compared with the baseline condition (*p* < 0.01, Figure 2C). On the other hand, metformin treatment decreased β-catenin protein levels compared with the basal condition only in A2780 cells (*p* < 0.05; Figure 2A. Supplementary Figure S5), but did not change β-catenin protein levels in HOSE or SKOV3 cells. Because the A2780 cell line was derived from a primary EOC [49], while SKOV3 cells are from ascites [50] (with elevated migration and invasion potential compared with A2780 cells [51]), these findings point towards differential responses of EOC cells to metformin treatment.

In addition, NGF increased the transcriptional activity of β-catenin/TCF-Lef (*p* < 0.05; Figure 2D,E), while metformin treatment blocked the NGF-mediated increase in transcriptional activity of β-catenin/TCF-Lef in EOC cells.

Both c-MYC and β-catenin/TCF-Lef regulate the expression of several proteins that are important in tumour development, including survivin and VEGF. Thus, in subsequent experiments, we evaluated the effects of NGF and metformin treatment on the levels of these proteins.

### 2.3. Metformin Decreases NGF-Induced Survivin Levels in Ovarian Cells

**Figure 3:**
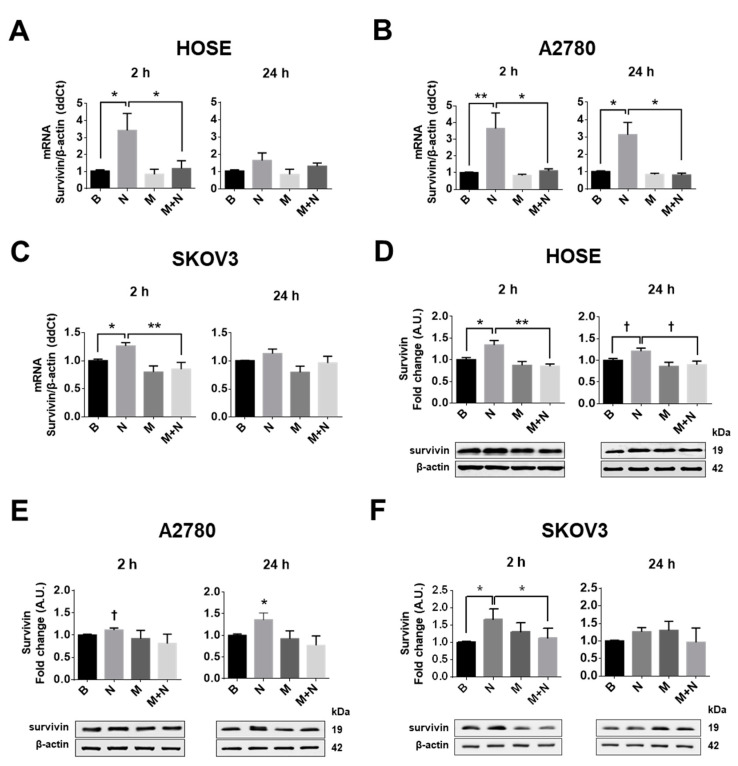
Metformin decreases the NGF-dependent increase in survivin expression in ovarian cells. Ovarian cells were treated with metformin 10 mM for 48 h and/or NGF 100 ng/mL or 150 ng/mL (A2780/HOSE cells and SKOV3 cells, respectively) for 24 h or the last 2 h. Then, survivin mRNA was detected in the ovarian cell lines HOSE, A2780 and SKOV3 by RT-PCR and protein levels were determined by Western blotting. (**A**–**C**) mRNA levels of survivin in the ovarian cell lines following metformin and NGF treatment. (**D**–**F**) Western blots of survivin in ovarian cell lines. *N* = 4 experiments per condition. * *p* < 0.05; ** *p* < 0.01 as indicated or with respect to basal condition. Statistical analysis: Kruskal–Wallis and Dunn´s post-test. ^†^ *p* < 0.05 with respect to basal condition or where indicated using the Mann–Whitney test. B: basal condition (without stimuli), N: NGF, M: metformin treatment. Results are expressed as the mean ± standard error of the mean (SEM).

NGF stimulation of ovarian cells increased mRNA levels of the anti-apoptotic protein survivin in all cell lines studied (*p* < 0.05; Figure 3A–C; Supplementary Figure S6). In addition, NGF increased survivin protein levels in ovarian cell lines, with a strong effect after 2 h of stimulation in HOSE and SKOV3 cells (*p* < 0.05; Figure 3D,F) and after 24 h of stimulation in A2780 cells (*p* < 0.05; Figure 2E). Metformin treatment did not decrease basal survivin levels. However, metformin treatment did block the NGF-mediated increase in survivin mRNA and protein levels in HOSE and SKOV3 cells (*p* < 0.01 and *p* < 0.05; Figure 3A–F, Supplementary Figure S7).

### 2.4. Metformin Decreases NGF-Induced VEGF Expression in Ovarian Cancer Cells

**Figure 4:**
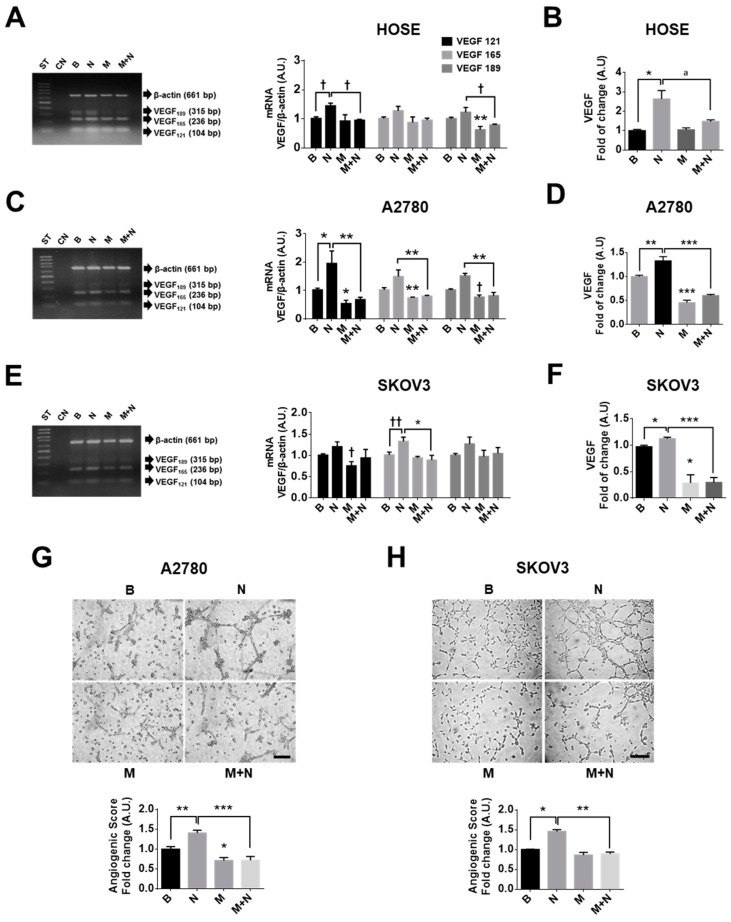
Metformin prevents the NGF induced VEGF expression and angiogenic properties of ovarian cells. Ovarian cells were treated with metformin 10 mM for 48 h and/or NGF 100 ng/mL or 150 ng/mL (A2780/HOSE cells and SKOV3 cells, respectively) for 24 h or the last 2 h, and then processed to detect VEGF expression. (**A**,**C**,**E**) Representative agarose gels with PCR products of different VEGF transcripts (VEGF 121, VEGF 165 and VEGF 189 amino acids) in ovarian cells. Semi-quantification of *N* = 4 or more independent experiments. (**B**,**D**,**F**) VEGF detection in the culture supernatants of ovarian cells by enzyme-linked immunosorbent assay (ELISA). *N* = 4 or more independent experiments induplicate. (**G**,**H**) Angiogenic score calculated as indicated in the methodology section with conditioned medium from A2780 or SKOV3 cells, respectively. Bar = 50 µm. *N* = 4 or more independent experiments (4–8 images were evaluated per experiment). * *p* < 0.05; ** *p* < 0.01 and *** *p* < 0.001 as indicated or with respect to basal conditions. Statistical analysis: Kruskal–Wallis and Dunn´s post-test. ^†^ *p* < 0.05 and ^††^ *p* < 0.01 with respect to basal conditions or as indicated using the Mann–Whitney test. B: basal condition (without stimuli), N: NGF, M: metformin treatment. Results are expressed as the mean ± standard error of the mean (SEM).

In the ovary, alternative splicing during the transcription of VEGF mRNA is responsible for generating different transcripts and protein isoforms [52]. The transcripts VEGF 121, VEGF 165 and VEGF 189 lead to VEGF peptides of 121, 165 and 189 amino acids in length of which VEGF 121 and 165 are the most abundant in EOC cells [8,53]. Our results showed that NGF increased the mRNA of VEGF 121 in non-tumour and EOC cells (*p* < 0.05). Moreover, the mRNA of VEGF 165 and 189 increased in EOC cells, whereby the greatest effect was observed at short time points (2 h of stimulation; *p* < 0.01; Figure 4A,C,D and Supplementary Figure S8). Furthermore, NGF stimulation increased VEGF levels in the culture supernatants of ovarian cells, whereby the greatest increase was seen after 2 h (*p* < 0.05 and *p* < 0.01; Figure 4B,D,E). On the other hand, metformin treatment decreased the mRNA of VEGF 189 in non-tumour ovarian cells (*p* < 0.01; Figure 4A) and the mRNA of VEGF 121, 165 and 189 in EOC cells (*p* < 0.05 and *p* < 0.01; Figure 4C,E). As expected, metformin treatment decreased VEGF levels in the culture supernatants of EOC cells compared with those detected under basal conditions (*p* < 0.05 and *p* < 0.001; Figure 4D,F), changes that were not observed in HOSE cells (Figure 4B). Importantly, co-treatment with metformin and NGF revealed that metformin blocks NGF-induced VEGF mRNA and protein levels in all cell lines (*p* < 0.05, *p* < 0.01 and *p* < 0.001; Figure 4A–F).

### 2.5. Metformin Decreases the Vasculogenic Potential of Ovarian Cancer Cells Induced by NGF

Subsequently, a functional assay using the conditioned medium from EOC cells (stimulated with NGF and/or metformin) was used to evaluate effects in the endothelial cell line EA.hy926. Conditioned media obtained after NGF stimulation of EOC cells (A2780 and SKOV3 cells) increased the angiogenic score of the EA.hy926 cells (*p* < 0.01; Figure 4G,H), while metformin treatment of EOC cells decreased the response (*p* < 0.05). Moreover, metformin treatment of EOC cells blocked the NGF-induced angiogenic score in endothelial cells (*p* < 0.001).

### 2.6. Metformin Increases Levels of miR-23b and miR-145 and Prevents the NGF-Induced Decreases of These miRs in EOC Cells

Both NGF and metformin have been suggested to modulate miRs abundance. Previous results from our group showed that oncosuppressor miR-23b and miR-145 are downregulated in EOC [35], which permits increased transcription of the mRNA targets. An in-silico analysis suggested that c-MYC and VEGF might be regulated by miR-23b and miR-145 (Table 1).

**Figure 5:**
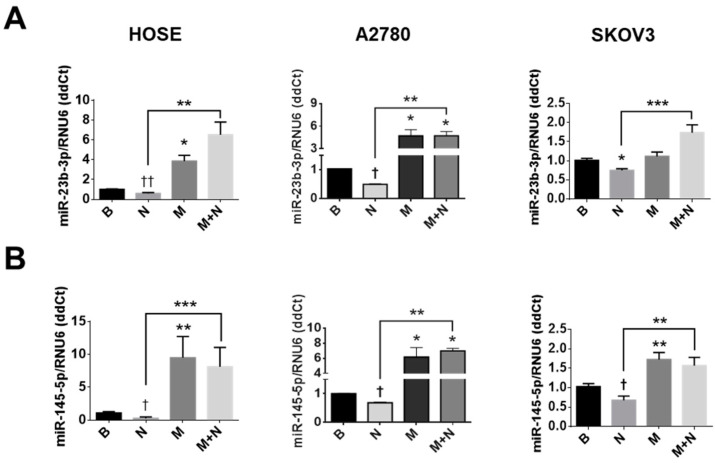
Metformin increases the expression of miR-145 and miR-23b in ovarian cell lines. Ovarian cells were treated with metformin (10 mM for 48 h) and NGF (100 or 150 ng/mL, last 3 h) to detect miR expression. miR-23b and miR-145 were quantified in HOSE, A2780 or SKOV3 cells by qPCR. *N* = 4 or more independent experiments in duplicate. * *p* < 0.05; ** *p* < 0.01 and *** *p* < 0.001 as indicated or with respect to basal conditions. Statistical analysis: Kruskal–Wallis and Dunn´s post-test. ^†^ *p* < 0.05 and ^††^ *p* < 0.01 with respect to basal conditions or where indicated using the Mann–Whitney test. B: basal condition (without stimuli), N: NGF, M: metformin treatment. Results are expressed as the mean ± standard error of the mean (SEM).

With this in mind, we assessed the effect of NGF and metformin on miR-23b and miR-145 levels in ovarian cell lines. As Figure 5 shows, NGF treatment decreased the levels of miR-23b (*p* < 0.05 for A2780 and SKOV3 cells; Figure 5A) and miR-145 levels (*p* < 0.05; Figure 5B) in EOC cells, while metformin increases miR-23b (*p* < 0.05 for HOSE and A2780 cells) and miR-145 levels in ovarian cell lines (*p* < 0.01, *p* < 0.05 and *p* < 0.01 for HOSE, A2780 and SKOV3 cells, respectively). In addition, the presence of metformin attenuated the decrease in miR-23b and miR-145 induced by NGF in the three cell lines (*p* < 0.01 and *p* < 0.001; Figure 5A,B).

## 3. Discussion

In the present study, we sought to shed light on the tumour suppressor mechanisms triggered by metformin in EOC cells. Previous in-vitro studies by our group showed that metformin blocks the pro-angiogenic and proliferative effects of NGF/TRKA [46]. In the current study we provide evidence that this effect can be attributed to a decrease in the expression of several important oncoproteins, including c-MYC, VEGF and survivin. In addition, a decrease in the transcriptional activity of c-MYC and β-catenin/TCF-Lef was observed in EOC cells. These changes coincide temporally with increased levels of miR-23b and miR-145 in ovarian cells, likely reflecting the reduced ability of NGF to decrease these oncosuppressor miRs in the presence of metformin. Our in-silico studies show that the proteins VEGF, c-MYC and survivin may represent miR-23b and miR-145 targets. Thus, the observed up-regulation of these miRs in the presence of metformin likely explains the changes in these proteins due to metformin treatment, as well as the pleiotropic effects of metformin described by other authors, given that one miR may potentially regulate the expression of hundreds of cell proteins [54,55].

EOC is the leading cause of death due to gynaecological neoplasia in developed countries and therapeutic success has not improved substantially in the last decades [2,3,56,57,58]. A better understanding of the molecular mechanisms underlying EOC is necessary in order to identify new therapeutic targets and develop new therapeutic strategies. The current results identify the use of metformin as a potentially attractive adjuvant therapy in the context of EOC.

A limitation of this study is that the metformin concentrations used here (10 mM, 48 h) were considerably higher than the plasma concentrations of this drug generally described in the literature [59]. However, bearing in mind the pharmacodynamics, as well as studies suggesting that metformin may accumulate in rodent tissues [60,61] it is possible that the elevated concentrations employed here are relevant. The concentration of metformin reached in tissues, like the ovary, is controversial; however, a recent study identified micromolar metformin concentrations in mouse ovarian cancer tumours [62]. Another limitation to our in-vitro experiments is that only relatively short time points were evaluated. In patients, metformin can accumulate in ovarian tissues, because patients consume this drug for extended periods of time. It is also for this reason that we decided to test the higher dose in our short term in vitro experiments.

In future experiments, it will be important to corroborate these in-vitro results in mouse models. There are several studies in which the anti-tumor effects of metformin have been evaluated in-vivo but, unfortunately, working with NGF is more difficult, because the half-life in circulation is only 2.3 h following intravenous injection [63]. For this reason, the use of mini-osmotic pumps is recommended to guarantee stable levels of circulating NGF for several days [63]. We plan to set up such experiments in the future.

**Figure 6:**
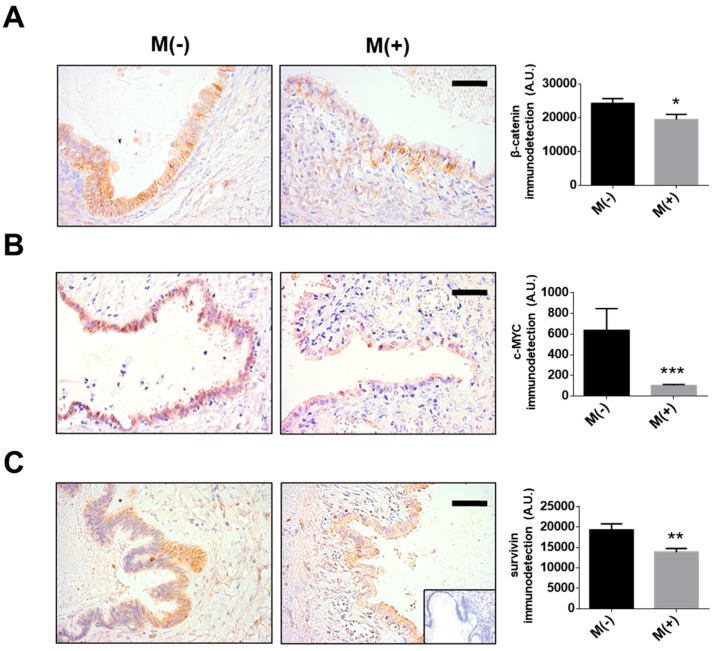
Metformin intake decreases the presence of β-catenin, c-MYC and survivin in epithelial ovarian tumours. Sequential paraffin-embedded samples from female users (M+) and non-users (M−) of metformin were used to detect oncoproteins by immunohistochemistry: β-catenin (**A**) c-MYC (**B**), or survivin (**C**) levels. *N* = 4 independent experiments (6–8 images were evaluated per experiment). Bar = 40 µm. * *p* < 0.05; ** *p* < 0.01 and *** *p* < 0.001 according to the Mann–Whitney test. Results are expressed as the mean ± standard error of the mean (SEM).

Given these limitations, we also compared β-catenin, c-MYC and survivin levels in samples from a small group of patients with serous and mucinous borderline ovarian tumours that were either users or not of metformin. The results are consistent with our in-vitro experiments. Specifically, in the biopsies from patients who had taken metformin levels of β-catenin, c-MYC and survivin were lower compared to those found in biopsies from patients that were not under treatment with this drug (see Figure 6). These findings support the notion that our in-vitro experiments represent an adequate proxy to define how mechanistically metformin acts in ovarian cancer patients. It is important to note that the proteins analysed by immunohistochemistry (IHC) were detected in the early stages of EOC development (borderline ovarian tumours). This because we only had access to a reduced number of ovarian cancer patients that were users of metformin. In any case, the results shown here are promising and indicate that metformin intake may serve to prevent/limit the progression of ovarian cancer, as suggested previously by others [39,64]. However, to consolidate these promising preliminary results, further patient-based studies involving many more patients will be necessary.

It is interesting to note that we worked here with three different cell lines, which represent different stages in epithelial ovarian cancer progression. HOSE cells are a well-known model of immortalized, but non-tumoral ovarian surface epithelial cells [7,65,66], while the A2780 cell line was obtained from primary ovarian adenocarcinoma of a patient without treatment [49], and finally the SKOV3 cell line was isolated from the ascites of a woman with EOC [50]. SKOV3 cells are resistant to several cytotoxic drugs and are highly migratory and invasive [51,67], similar to metastatic cells. Not surprisingly, some notable differences in the pattern of responses to metformin were apparent. For instance, in HOSE cells the oncoproteins studied were essentially not affected by metformin treatment, while A2780 cells were the most sensitive to metformin treatment. Unfortunately, SKOV3 cells were less sensitive to metformin than A2780 cells. Moreover, metformin increased the oncosuppressor miR-23b and miR-145 by five times or more in A2780 cells, while in SKOV3 cells, levels of miR-145 increased only 1.7 times. These findings indicate that SKOV3 cells may be more resistant to metformin treatment and that the therapeutic benefit of metformin might be temporarily limited. However, other reports showed that metformin reduced the migration and invasiveness of SKOV3 cells [68,69] and we were able to replicate these findings using our experimental conditions in vitro (Supplementary Figure S9). These observations may be taken to suggest that metformin has more pronounced effects on the behaviour of EOC cells with elevated metastatic potential.

Moreover, we provide evidence that NGF/TRKA stimulation decreases the levels of the oncosuppressors miR-145 and miR-23b, but the magnitude of the NGF effect varies between the EOC cell lines A2780 and SKOV3. These differences may relate to the baseline levels of the miRs. For example, miR-145 is considered a suppressor of cell migration and invasion [70] and the levels of miR-145 are lower in SKOV3 cells than A2780 cells (Supplementary Figure S9). This may explain why SKOV3 cells are more migratory and display an elevated metastatic potential compared to A2780 cells [51,71]. In addition, the low basal levels of miR-145 likely explain why NGF stimulation leads to only a moderate decrease in the levels of this miR in SKOV3 cells. Alternatively, A2780 cells express higher basal levels of miR-145 and in these cells NGF induced a strong decrease in miR-145 content. Additionally, the differences in the responses of EOC cells to NGF stimulation may also reflect differential expression of neurotrophins and their receptors. For instance, Pro-NGF also promotes signalling by binding directly to TRKA or P75 (low affinity receptor of NGF)/sortilin in breast cancer and melanoma cells [72,73]. As shown in Supplementary Figure S10, NGF levels are lower in SKOV3 cells than A2780 cells. On the other hand, Pro-NGF levels are higher in SKOV3 cells. These results are in agreement with a previous study [74], which described that SKOV3 cells express less NGF and TRKA receptor, but elevated protein levels of the P75 receptor compared with A2780 cells. These findings could explain why SKOV3 cells required higher concentrations of NGF (150 ng/mL vs. 100 ng/mL used in A2780 cells) to increase proliferation/migration, given that SKOV3 cells express fewer TRKA receptors. Furthermore, the higher levels of Pro-NGF and the P75 receptor in SKOV3 cells may explain why SKOV3 cells migrated more and displayed an elevated metastatic potential compared to A2780 cells.

Another possible limitation of this study is that the ovarian cell lines used are not representative of high grade serous (HGS) ovarian carcinoma [75], which is described as the most malignant form of the disease. However, a recent report shows that non-HGS EOC cell lines (as A2780 and SKOV3) migrate and invade to a greater extent than those derived from HGS carcinomas [51], suggesting that these non-HGS cells may in fact have a higher metastatic potential than cells derived from HGS carcinomas. Importantly, our results show that metformin reduces significantly the migration and invasion of these aggressive cell lines. In addition, metformin has been shown to improve the chemosensitivity of ovarian cancer cells with a resistant phenotype [76]. In this case, miR modulation may provide a possible explanation. For instance, miR-145 is known to regulate several transporters of the ABC1 family and SLC1A2 [77,78], which are important regulators of drug entry in cancer cells. Thus, the results presented here open up many interesting possibilities concerning the mode of metformin action that merit more detailed analysis in the future.

Another interesting point is that metformin treatment increased miR-145 and miR-23b in all the ovarian cells used, including HOSE cells. That may be because HOSE are not entirely normal, but rather immortalized epithelial ovarian cells [66]. HOSE cells are significantly different from EOC cells and they are considered as non-tumoral model or used as a model of early stages of tumoral development [7,65,66]. That said, it is highly likely that they have different sets of miRs compared to primary epithelial ovarian cells.

AKT and ERK signalling pathways activated by NGF/TRKA (see Supplementary Figure S5) favour cancer cell growth [7]. Specifically, β-catenin translocation and β-catenin/TCF-Lef transcriptional activity are associated with AKT activation and positive feedback loops have been shown to connect these components; for instance, survivin and β-catenin/TCF-Lef promote AKT signalling in cancer cells [25]. Alternatively, ERK activation is associated with an increase in c-MYC protein levels and its transcriptional activity, as well as an increase in VEGF levels in several cancer models [11,12,13,14,15]. c-MYC appears to be a key target of metformin, which could explain several of the anti-tumor effects observed. c-MYC can increase the transcription of the β-catenin and VEGF genes, and, at the same time, β-catenin/TCF-Lef activity depends on c-MYC transcription in colorectal cells [79], suggesting that all these proteins collaborate in signalling loops to favour the development and progression of cancer cells.

Additionally, decreased expression of oncosuppressor miRs has been reported as the consequence of AKT/ERK signalling. For example, in bladder cancer, ERK-mediated activation by the EGFR decreases miR-23b [80]. In addition, in breast cancer cells, the activation of AKT down-regulates miR-145 levels [81]. These findings coincide with our current results in EOC cells. Interestingly, previous results from our group showed that miR-23b and miR-145 are downregulated in ovarian cancer samples [35]. Here we observed that NGF stimulation decreased the levels of both miRs in ovarian cells, while metformin treatment had the opposite effect. Recent studies have shown that metformin increases the expression of the endoribonuclease Dicer [82] in hepatocellular carcinoma cells, which likely increases the production of some oncosuppressor miRs that are downregulated in cancer cells. Our present results indicate that such mechanisms may also be in place in EOC cells. However, more work in future studies is required to corroborate this possibility.

An interesting point is that metformin blocks the tumour-promoting effects of NGF, but these apparently do not depend on decreased AKT signalling. With respect to this point, it should be noted that AMPK-activation by metformin triggers the unfolded protein response (UPR)-mediated cell death with a compensatory activation of AKT in lymphoblastic leukaemia lymphoblasts [83]. We obtained comparable results in EOC cells, where metformin treatment induces AKT signalling, as shown in Supplementary Figure S11. Metformin treatment did not decrease AKT phosphorylation in the amino acid residue threonine 308, a key phosphorylation site implicated in AKT activation by PI3K [84]. In addition, our results showed that metformin increased the phosphorylation in serine 473, a substrate for mammalian target of rapamycin 2 (mTORC2) [85]. Metformin is a known inhibitor of mTORC1 and selective mTORC1 inhibition can lead to mTORC2 and AKT activation as a compensatory response [85]. This mechanism may explain how EOC cells become resistant to metformin treatment. Another important point is that metformin treatment increases AKT ubiquitination and degradation, which coincides with the reduced AKT levels observed in our experimental conditions (Supplementary Figure S11A–F). However, we did observe that metformin treatment blocked the increase in ERK activation by NGF (Supplementary Figure S11G). So, anti-proliferative effects and miR upregulation by metformin may be attributable to the decrease in ERK activation observed in EOC cells.

Another key point is that the combination of metformin + NGF might have adverse effects, possibly as the consequence of excessive reactive oxygen species (ROS) accumulation in EOC cells, which is known to promote cell cellular senescence and cell death [86]. The use of metformin in millimolar concentrations is known to produce a significant increase in ROS in human breast cancer cells [87], hepatocellular carcinoma [88] and colorectal cancer cells [89]. In addition, NGF reportedly also increases ROS production in neuronal cells [90]. So, such excessive ROS accumulation in the groups M + N may have reduced responses below what was to be expected in some of the assays we report on here.

Interestingly, survivin levels were not affected by metformin treatment in EOC cells. However, c-MYC and β-catenin (that control survivin transcription) levels decreased following metformin treatment. This may again point towards the presence of adaptive mechanisms in EOC cells that favour resistance to metformin treatment. For instance, survivin mRNA may possess aberrant alternative polyadenylation sites that prevent inhibition by miRs and thus increase survivin expression [91].

With respect to the anti-angiogenic proprieties of metformin, a key finding here is that metformin decreased VEGF levels. This point is relevant because one characteristic of EOC is the high degree of angiogenesis. The enzyme-linked immunosorbent assay (ELISA) kit used in this study recognises the VEGF isoforms 121 and 165 (secreted VEGF) and our results showed that metformin treatment decreased all three VEGF transcripts in ovarian cells (VEGF 121, 165 y 189). This finding is consistent with the functional experiments performed using endothelial cells in a matrigel assay. There, conditioned medium from EOC cells treated with metformin decreased the angiogenic score of EA.hy926 endothelial cells, showing that metformin not only reduced VEGF expression but also the angiogenic potential of conditioned media from EOC cells.

**Figure 7:**
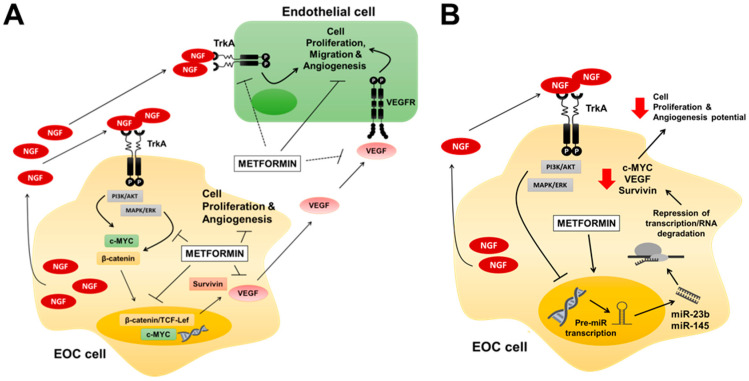
Proposed mechanisms by which metformin prevents NGF–mediated effects in EOC cells. (**A**) Metformin blocks the NGF-induced c-MYC, survivin and VEGF protein levels, decreasing the proliferative and angiogenic potential of EOC cells. NGF and VEGF are depicted as acting directly on endothelial cells, while c-MYC and survivin changes contribute to EOC cell proliferation and VEGF expression. (**B**) Metformin increases the expression of oncosuppressor miR-145 and miR-23b, which may be responsible for the observed decreases in c-MYC, survivin and VEGF levels in EOC cells. Filled arrows mean stimulation, thick perpendicular bars mean inhibition and dotted lines mean indirect association.

In summary, the present study sheds light on some mechanisms by which metformin prevents the tumour-promoting effects of NGF in EOC cells. These involve the decrease in the presence of oncoproteins, such a c-MYC and VEGF, as well as the transcriptional activity of MYC and β-catenin-TCF-Lef, which were paralleled by an upregulation of miR-23b and miR-145 following metformin treatment (Figure 7). Taken together, these results strongly suggest that metformin should be considered as a future alternative in the treatment of EOC. As suggested by a reviewer of this manuscript, additional data related to EOC biopsies will be necessary in order to corroborate in vitro findings, but due to the COVID-19 situation, such experiments had to be delayed and preliminary results were presented in this communication.

## 4. Material and Methods

### 4.1. Cell Culture and Treatments

Human ovarian surface epithelium (HOSE) cells were donated by Dr. David Munroe (National Cancer Institute, National Institute of Health, Bethesda, MD, USA). The human epithelial ovarian cancer cell line A2780 was obtained from the European Collection of Authenticated Cell Cultures (ECACC) and SKOV3 cell line was obtained from the American Type Culture Collection (ATCC). A2780 and HOSE cells were cultured in Dulbecco’s minimal essential medium/Ham F-12 without phenol red (Sigma-Aldrich Co, St Louis, MO, USA). SKOV3 cells were cultured in Roswell Park Memorial Institute (RPMI) 1640 medium (Life Technologies, Thermo Fisher Scientific, Waltham, MA, USA). Culture medium was supplemented with 2% fetal bovine serum (FBS) and penicillin/streptomycin (100 µg/mL and 100 U/mL). Cells (500,000) from all ovarian lines were treated with metformin chlorhydrate 10 mM (Sigma-Aldrich Co) for 48 h and NGF 100 ng/mL (Sigma-Aldrich Co) for 24 h or the last 2 h. EA.hy926 cells (human endothelial cell line) were maintained in Iscove’s Modified Dulbecco’s Medium (Thermo Fischer Scientific, Waltham, MA, USA) supplemented with 10% FBS. Prior to use, these cells were grown in serum deprived medium for 24 h and then stimulated with culture supernatants from EOC cells for another 8 h. The NGF and metformin concentrations were chosen according to dose–response curves established in previous experiments [10,46].

### 4.2. Tissue Samples

Ovarian tissues were obtained from patients in the Hospital Clínico Universidad de Chile that signed an informed consent form approved by the Institutional Ethics Committee (Record N° 022, 2016). Recruited women completed a survey and were segregated into users and non-users of metformin. Sequential Paraffin-embedded samples, classified as epithelial serous or mucinous borderline tumours (*N* = 8) by an expert pathologist, were used for immunohistochemistry (IHC) analysis.

### 4.3. Protein Detection by Western Blotting

Protein extracts (30 or 50 µg of total protein) were separated by SDS-polyacrylamide gel electrophoresis (SDS-PAGE) and transferred to nitrocellulose membranes. The membranes were then incubated with anti-survivin (R&D Systems, Minneapolis, MN, USA, #AF886; 1:3000 for A2780/SKOV3 and 1:1000 for HOSE cells), anti-c-MYC (Cell Signaling, Danvers, MA, USA, #5605, 1:500) or anti-β-catenin antibody (BD Transduction Laboratories, Franklin Lakes, NJ, USA, #610154,1:3000 for A2780/SKOV3 and 1:1000 for HOSE cells) at 4 °C overnight. β-actin (Sigma-Aldrich Co, #A2228, 1:10,000) was used as loading control.

### 4.4. Immunohistochemistry

Immunohistochemistry (IHC) was performed on tissue samples as previously described [8]. Anti-survivin (R&D Systems #AF886; 1:500), anti-c-MYC (Abcam, Cambridge, UK, ab32072, 1:500) and anti-β-catenin antibodies (BD Transduction Laboratories, #610154, 1:1000) were incubated at 4 °C overnight. For each slide, a negative control incubated only with 2% PBS-BSA without the primary antibody was included. Five to ten microphotographs were obtained with each sample. Integrated optical density (IOD) measurements processed using the computer program Image-ProPlus 6.2 (Media Cybernetics Inc., Silver Spring, MD, USA) provided the data for semi-quantification of antigen levels.

### 4.5. Immunocytochemistry

Immunocytochemistry (ICC) experiments were performed as previously described [46].c-MYC (Cell Signaling #5605, 1:500) and β-catenin (BD Transduction Laboratories, #610154, 1:1000) were detected and IOD values were obtained as described above.

### 4.6. VEGF Determination

VEGF levels in culture supernatants were determined with an enzyme immunosorbent assay (ELISA) Kit (R&D Systems, #DVE00), according to the manufacturer’s instructions.

### 4.7. Total RNA Extraction and RT-PCR

Total RNA was extracted using the phenol-chloroform method. RNA (2 µg) was used for reverse transcription, as described previously [92]. VEGF and β-actin cDNA were amplified by traditional PCR using GoTaq flexi polymerase (Promega, Madison, WI, USA). Survivin mRNA levels were assessed by real time PCR using Brilliant II SybrGreen QPCR master mix (Agilent Technologies, Santa Clara, CA, USA). All primers used are described in Supplementary Table S1. β-actin expression was used as invariant control gene. Sterile water instead of cDNA was added as a negative control for the reactions.

### 4.8. β-Catenin/Tcf-Lef Reporter Assay

Cells (450,000) were transfected using 0.3 µL of ViaFect (Promega) in 50 µL of opti-MEM I reduced serum media (Thermo Fisher Scientific) and 0.5 µg of the following plasmids: (1) pTOP-FLASH (that contains β-catenin/TCF-Lef responsive elements), (2) pFOP-FLASH (non-inducible reporter construct), both donated by Dr. Hans Clevers (Hubrecht Laboratory, Uppsalalaan, The Netherlands) and (3) pPON-FLASH (β-galactosidase transfection control) donated by Dr. Sergio Lavandero (Facultad de Ciencias y Quimicas Farmaceuticas, Universidad de Chile), which were used as previously described [93]. After transfection cells were cultured for 8 h. Then, cells were deprived of FBS overnight and next morning stimulated. Cells were then lysed and luciferase activity was measured with the Dual luciferase reporter assay system (Promega, Madison WI, USA), according to the manufacturers’ instructions. LiCl (15 mM) was used as a positive control. β-galactosidase activity of constructs was measured as previously described [93].

### 4.9. MYC Reporter Assay

Cells (30,000) were analysed per experiment in 24-well plate using the CignalMyc Reporter Assay Kit (Qiagen, Hilden, Germany), according the manufacturer’s instructions. Because c-MYC expression is dependent on cell density, all the experiments were performed with cells at 70–80% confluency in serum-deprived medium.

### 4.10. miR Extraction and qPCR

miRs were extracted using Quiazol and the miRNeasy mini kit (Qiagen) according to the manufacturer’s instructions. For reverse transcription of RNA, the miScript II RT kit (Qiagen) was employed. For real time PCR, diluted cDNA (1/10) and the miScript SYBR Green qPCR (Qiagen) kit were used to assess mRNA abundance using the StepOne Real-Time PCR kit (Applied Biosystems, Foster City, CA, USA). All primers and the PCR program are described in Supplementary Table S2. RNU6 was used as housekeeping miR and bi-distilled water was used instead of cDNA in the negative control.

### 4.11. Invasion Assays

EOC cells (500,000) were serum-deprived for 24 h and stimulated with NGF and/or metformin for 2 h. Then, cells were detached and counted. A total of 30,000 cells were re-suspended in 300 µL of the corresponding conditioned medium per well of the BioCoat Matrigel invasion chambers (Corning Inc., Corning, NY, USA, #354480) that had previously been rehydrated according manufacturer’s instructions. The inserts were incubated at 37 °C for 24 h (A2780 cells) or 16 h (SKOV3 cells) and then cells were fixed in 500 µL of cold methanol (−20 °C) for 2 min and stained with 500 µL of 1% Toluidine blue. Cells attached to the lower membrane surface were counted. Inserts were photographed (4 or more images in each experimental condition at 200x magnification) and analysed using cell counter plugin of Fiji Image J program.

### 4.12. Vasculogenesis Assays

EA.hy926 cells (10,000) were serum-deprived for 24 h and then placed in 24-well plates coated with 150 µL of serum and phenol red-free matrigel (Corning, New York, USA) and 400 µL of the different conditioned media from A2780/SKOV3 cells. After 8 h, cells were photographed and the angiogenic score was calculated as previously described [94]. This index is based on the morphology of and the connections between endothelial cells and weighed by a factor of 0, 1 or 2, depending on the width of the walls of the polygonal structures formed [94]:

Angiogenic Score = (N° of sprouts + (N° of connected cells) × 2+ (N° of polygons) × 3)/(N° of total cells) + 0, 1 or 2

### 4.13. Statistical Analysis

Results were processed with Kruskal–Wallis Test followed either by the Dunn, or the Mann–Whitney post-test, as appropriate. Results were expressed as the mean ± standard error of the mean (SEM). A value of *p* < 0.05 was considered significant. Data analysis was carried out with the GraphPad Prism 6 Program.

## 5. Conclusions

Metformin blocks the tumour-promoting effects of NGF in EOC cells modulating several oncoproteins such as VEGF, c-MYC and survivin, as well as c-MYC and β-catenin/TCF-Lef transcriptional activity. In part, the pleiotropic effects of metformin could be mediated by post-transcriptional mechanisms involving miR-23b and miR-145 upregulation. Because therapies for EOC have yielded modest results and biopsies from patients who take metformin present decreased levels of the oncoproteins studied, metformin could be considered as a possibility to complement current treatments of EOC.

## Figures and Tables

**Table 1:** In-silico analysis of different online databases identified potential microRNA (miR)-145 and miR-23b pairing sequences in mRNAs of the following oncoproteins: vascular endothelial growth factor (VEGF) (VEGFA, the most common isoform overexpressed in cancer cells), c-MYC, survivin (BIRC5) and β-catenin (CTNNB1).

Data Base	miR-145	miR-23b
Microrna.org	BIRC5, VEGFA, c-MYC	CTNNB1, VEGFA
Targetscan	BIRC5	-
TarBase v.8	CTNNB1	CTNNB1, VEGFA, BIRC5
miRtarBase	c-MYC, VEGFA	-
MirWalk	c-MYC, VEGFA	-

## Supplementary Materials

The following are available online at https://www.mdpi.com/1424-8247/13/10/315/s1, Figure S1: Effect of NGF and metformin on the cell count and viability of SKOV3 cells, Figure S2: Metformin prevents the NGF-induced proliferation of SKOV3 cells, Figure S3: Representative images of c-MYC immunodetection in ovarian cells, Figure S4: NGF activates AKT and ERK signaling pathways in a TRKA dependent manner, Figure S5: Representative images of β-catenin immunodetection in ovarian cells, Figure S6: NGF increases survivin mRNA in ovarian cells, Figure S7: Representative images of survivin immunodetection in ovarian cells, Figure S8: Effect of NGF and metformin on the mRNA levels of VEGF in ovarian cells, Figure S9: Metformin prevents the increase in migration and invasion stimulated by NGF in EOC cells, Figure S10: Immunodetection of NGF and Pro-NGF in EOC cells, Figure S11: Metformin prevents ERK activation by NGF in EOC cells, Table S1: Primers sequence using traditional and real time PCR, Table S2: Primers and PCR program employed for miR detection.
