# Supplementary material for: Metformin Reduces NGF-Induced Tumour Promoter Effects in Epithelial Ovarian Cancer Cells
Source: Pharmaceuticals (Basel). 2020 Oct 16;13(10):315. doi: 10.3390/ph13100315 (PMC7602813; doi:10.3390/ph13100315)
Supplement: Supplementary file 1 [file pharmaceuticals-13-00315-s001.pdf]

Supplementary material

Metformin reduces NGF-induced tumour promoter effects in Epithelial Ovarian Cancer cells

	Primers	Application	Amplicon	Sequence
Survivin	Sense: 5´-CTG GCA GCC CTT TCT CAA GGA-3´<br>Antisense: 5´-GCA ACC GGA CGA ATG CTT TT-3	Real-time PCR	225 pb	NM_001168.2
β-actin	Sense: 5´- TGGCAC CCA GCA CAA TGA AGA -3´<br>Antisense: 5´- GAA GCA TTT GCG GTG GAC GAT -3´	Real-time PCR	166 bp	NM_001101.4
VEGF	Sense: 5´- AGG CCA GCA CAT AGG AGA GA -3´<br>Antisense: 5´-ACC GCC TCG GCT TGT CAC AT-3´	Traditional PCR	104, 236 and 315 bp	NM001025367, 8 and 9
β-actin	Sense: 5´-TGA CGG GGT CAC CCA CAG TGT GCC CAT CTA-3´<br>Antisense: 5´-CTA GAA GCA TTG CGG TGG ACG ATG GAG GG-3´	Traditional PCR	660 bp	NM_001101.4

Supplementary Table 1: Primers sequence using to traditional and real time PCR. VEGF: vascular endothelial growth factor. Bp: base pair.

<table>
<tr><th></th><th>Primers</th><th>PCR program
Tº and time</th></tr>
<tr><td>miR-23b</td><td>miR-23b_2 miScript Primer Assay (MS00031647, Qiagen)</td><td rowspan="3">94ºC 15 sec
55ºC 30 sec
70ºC 30 sec
40 cycles</td></tr>
<tr><td>miR-145</td><td>miR-145_1 miScript Primer Assay (MS00003528, Qiagen)</td></tr>
<tr><td>U6 small nuclear RNA (RNU6)</td><td>RNU6 miScript Primer Assay (MS00033740, Qiagen)</td></tr>
</table>

Supplementary Table 2: Primers and PCR program employed for miR detection.

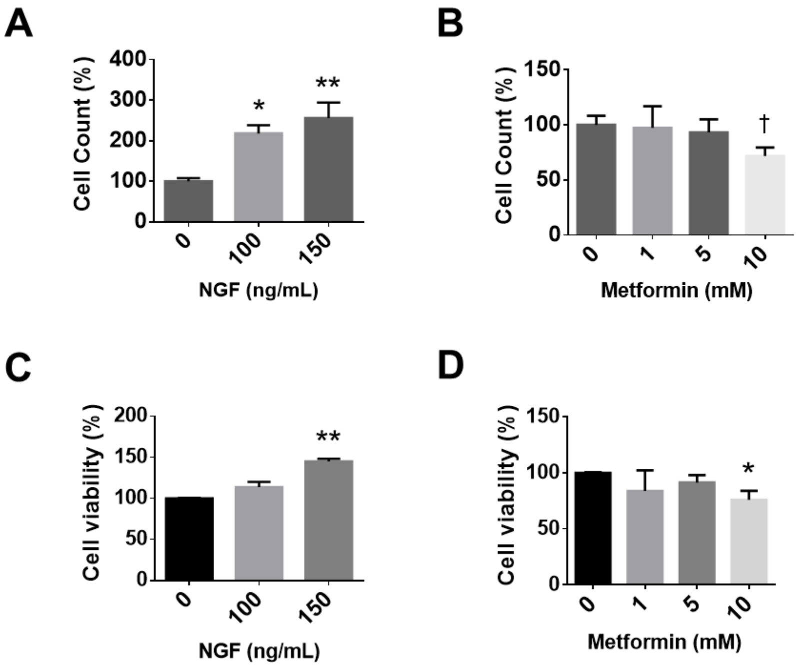

Supplementary Figure 1. Effect of NGF and metformin on the cell count and viability of SKOV3 cells. SKOV3 cells were incubated with NGF (0, 100 and 150 ng/mL) and metformin (0, 1, 5 and 10 mM) for 48 h and then cells were counted (**A, B**) and cell viability tests were performed (**C, D**). N=4 in triplicate. *=$p<0.05$ and **=$p<0.01$ with respect to the basal condition (Kruskal Wallis test and Dunn`s post-test). †=$p<0.05$ with respect to basal condition using the Mann Whitney test. Results are expressed as the mean ± standard error of the mean (SEM).

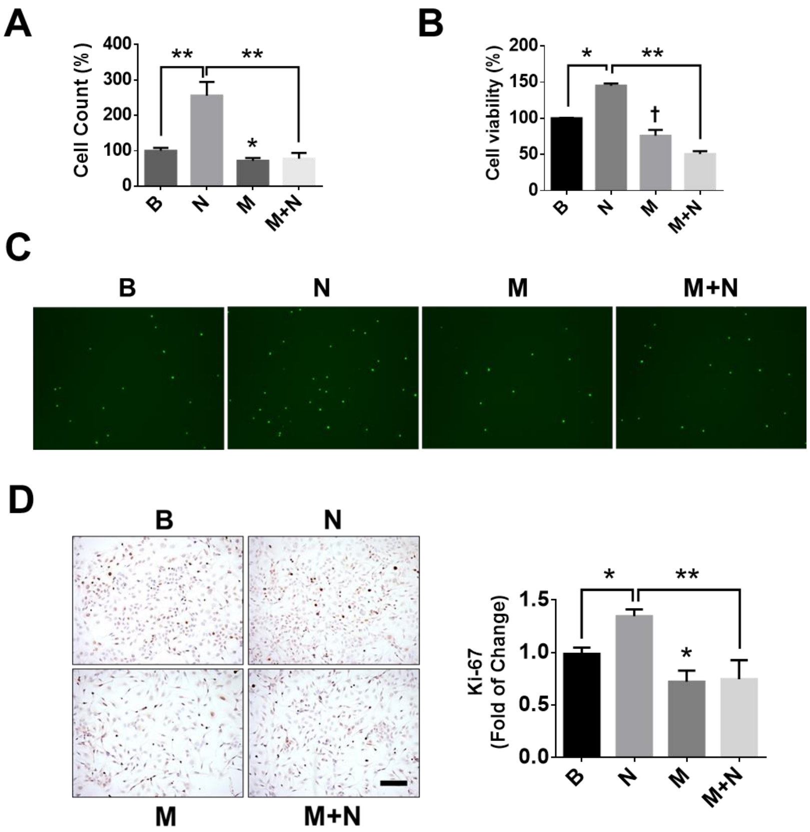

Supplementary Figure 2. Metformin prevents the NGF-induced proliferation of SKOV3 cells. (A, B) Cells were stimulated with metformin (10 mM) and NGF (150 ng/mL) for 48 h to evaluate cell number and cell viability. N=4 in triplicate. **(C)** Representative images of SKOV3 cells after treatments (stain: acridine orange that identifies only viable cells). **(D)** SKOV3 cells were treated with metformin for 48 h in the absence or presence of NGF for the last 6 h. Then cells were stained for presence of the ki-67 proliferation marker. Bar= 100 µm. N=4 (8 images per experiment were evaluated). *=p<0.05 and **=p<0.01 with respect to the basal condition or as indicated (Kruskal Wallis test and Dunn`s post-test). †=p<0.05 respect to basal condition using the Mann Whitney test. Results are expressed as the mean ± standard error of the mean (SEM).

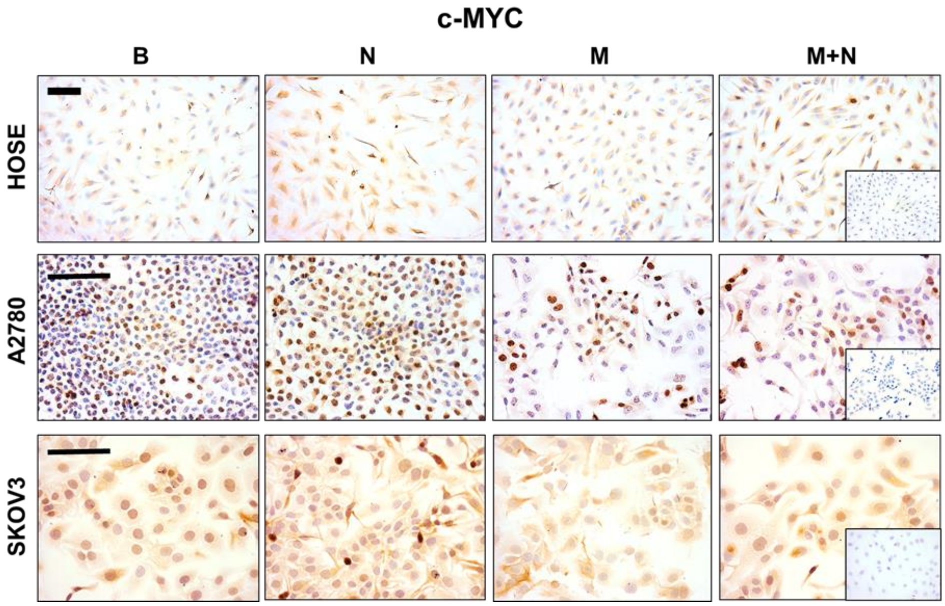

Supplementary Figure 3. Representative images of c-MYC immunodetection in ovarian cells. Cells were stimulated with metformin (M, 10 mM, 48 h) and/or NGF (N, 100 or 150 ng/mL last 2 h) and c-MYC immunocytochemistry was performed in HOSE, A2780 and SKOV3 cells. B: baseline condition (without stimuli). Bar=100 µm. Right lower insert: negative control (cells without primary antibody).

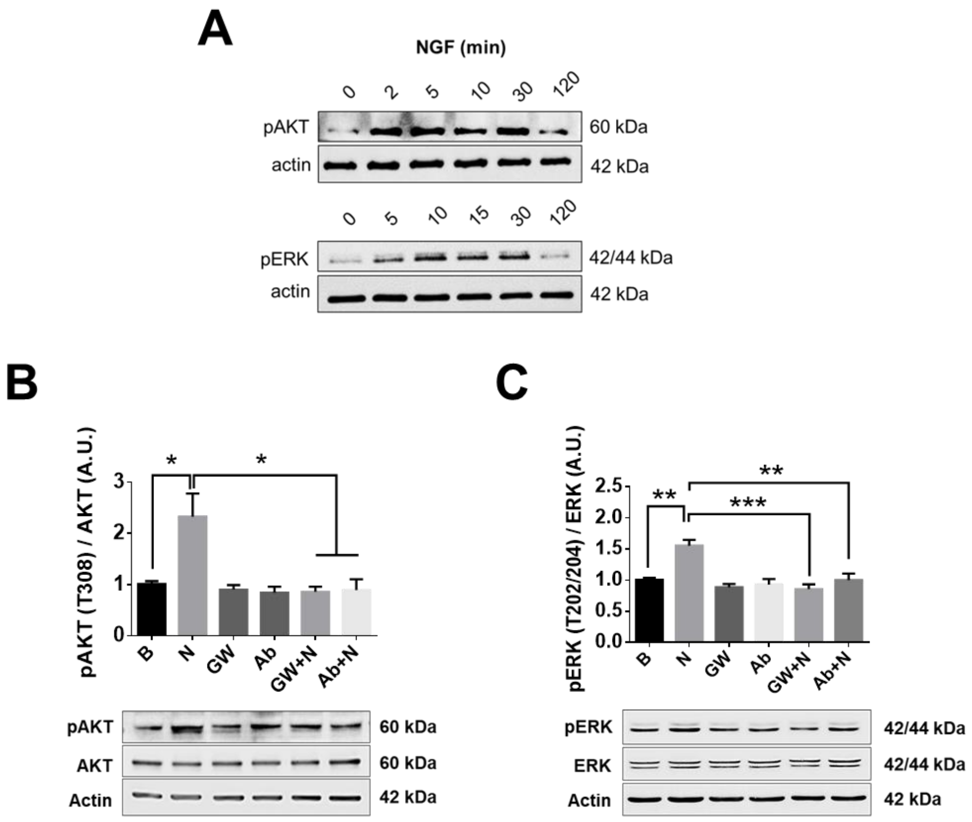

Supplementary Figure 4. NGF activates AKT and ERK signalling pathways in a TRKA dependent manner. A: A2780 cells were stimulated with NGF for 0, 2, 5, 10, 15, 30 and 120 min. Phospho-AKT (pAKT, S473) and phospho-ERK (pERK, T202/T204) levels were determined by western blotting after separating total protein extracts (50 ug). **B, C:** A2780 cells were treated with the specific TRKA inhibitor GW441756 (GW, 20 nM, Tocris Bioscience, Bristol, UK) or a neutralizing antibody (Ab, 5 µg/ml, Abcam, Cambridge, UK) for 1 h and then for the last 5 min stimulated with NGF (N, 100 ng/mL). B: baseline condition (without stimuli). N=4 or more independent experiments. *=p<0.05, **=p<0.01 and ***=p<0.001 as indicated (Kruskal Wallis test and Dunn`s post-test). Results are expressed as the mean ± standard error of the mean (SEM).

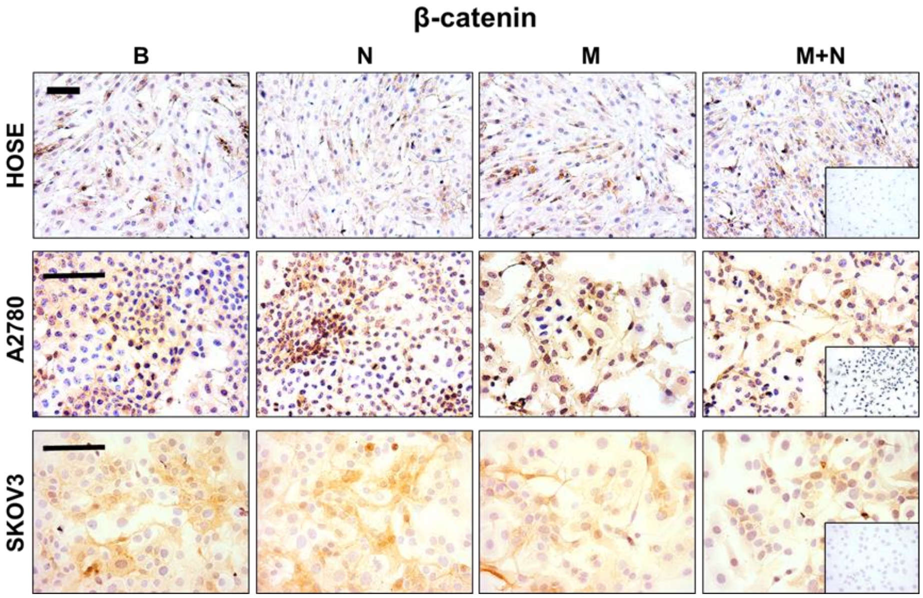

Supplementary Figure 5. Representative images of β-catenin immunodetection in ovarian cells. Cells were treated with metformin (M, 10 mM, 48 h) and NGF (N, 150 ng/mL last 2 h) and β-catenin staining was determined in HOSE, A2780 and SKOV3 cells. B: baseline condition (without stimuli). Bar= 100 µm. Right lower insert: negative control (cells without primary antibody).

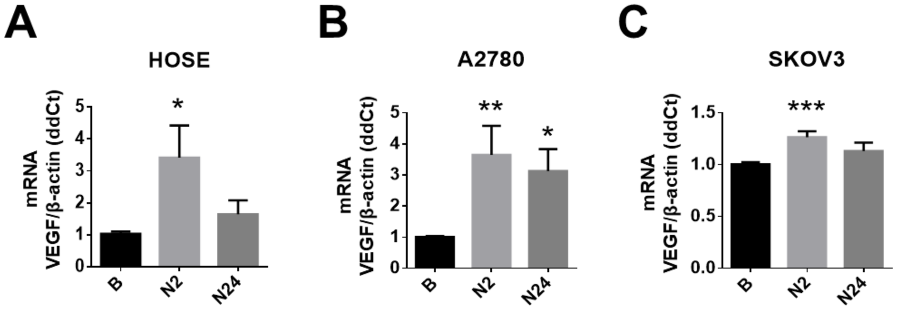

Supplementary Figure 6. NGF increases survivin mRNA in ovarian cells. HOSE **(A),** A2780 **(B)** and SKOV3 **(C)** cells were stimulated with NGF (100 or 150 ng/mL) for 2 (N2) or 24 h (N24) and survivin mRNA levels were determined by qPCR. B: baseline condition (without stimuli). N=4 or more experiments in duplicated. *=p<0.05 and **=p<0.01 with respect to the basal condition (Kruskal Wallis test and Dunn`s post-test). Results are expressed as the mean ± standard error of the mean (SEM).

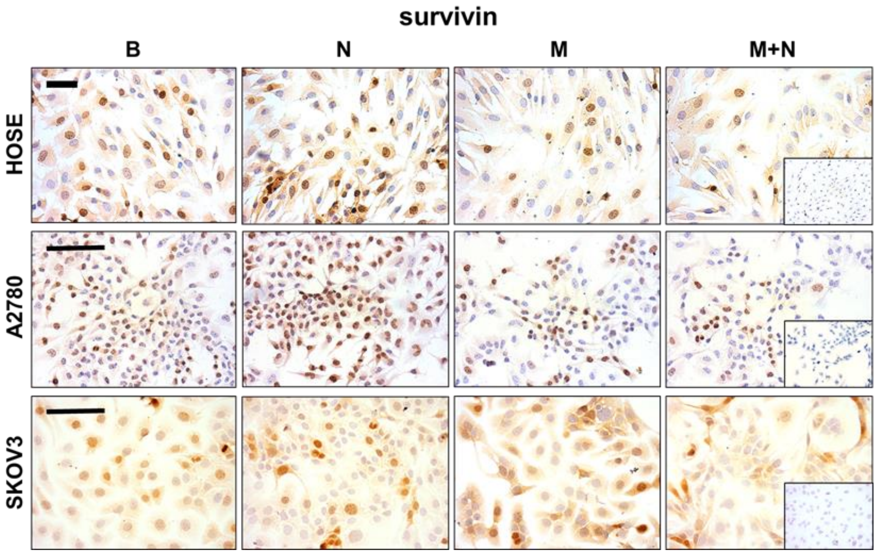

Supplementary Figure 7. Representative images of survivin immunodetection in ovarian cells. Cells were stimulated with metformin (M, 10 mM, 48 h) and NGF (N, 150 ng/mL last 2h) and survivin staining was determined in HOSE**,** A2780 and SKOV3 cells. B: baseline condition (without stimuli). Bar= 100 µm. Right lower insert: negative control (cells without primary antibody).

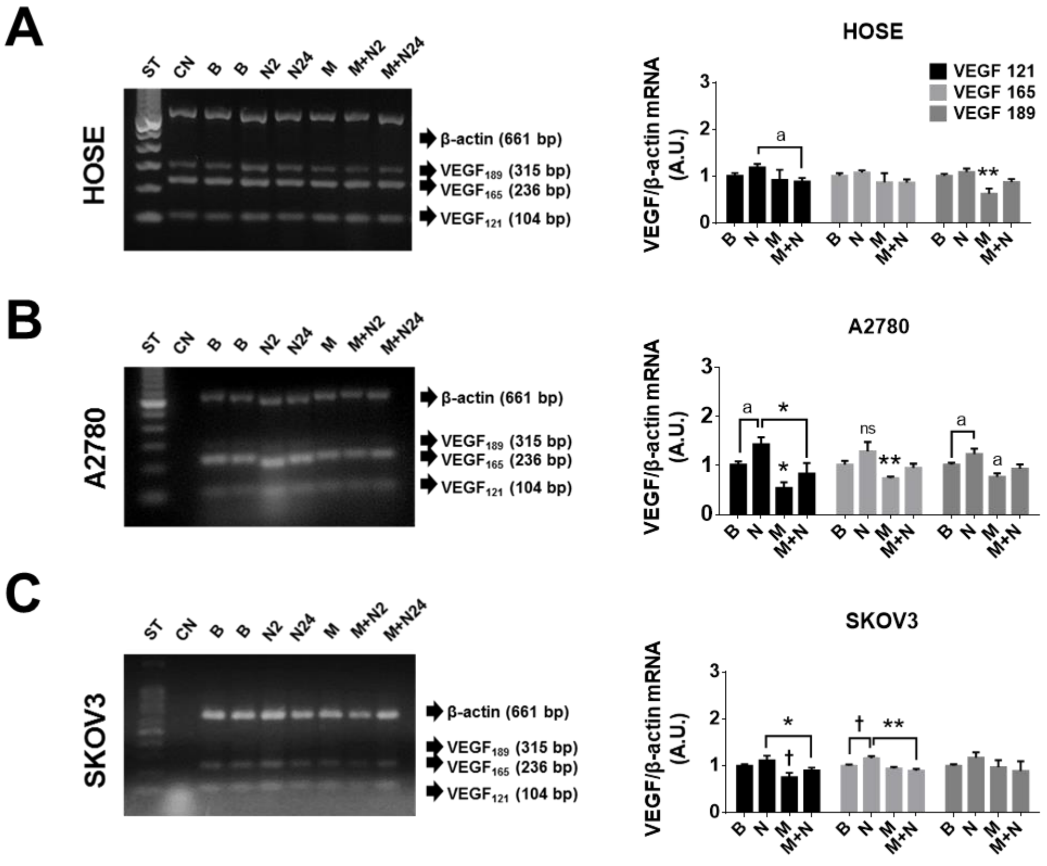

Supplementary Figure 8. Effect of NGF and metformin on the mRNA levels of VEGF in ovarian cells. Ovarian cells HOSE **(A)**, A2780 **(B)** and SKOV3 **(C)** were stimulated with metformin (10 mM, 48 h) and NGF (150 ng/mL, the last 2 or 24 h) and the main three transcripts of VEGF (121, 165 and 189 aminoacids) were analysed by conventional PCR. B: baseline condition (without stimuli). N2: NGF for 2 h. N24: NGF for 24 h. M: metformin. The semi-quantification shown to the right corresponds to results obtained after 24 h of stimulation with NGF. N=4 independent experiments. *=p<0.05 and **=p<0.01 with respect to basal conditions or as indicated (Kruskal Wallis test and Dunn`s post-test). †=p<0.05 with respect to the basal conditions or as indicated using the Mann Whitney test. Results are expressed as the mean ± standard error of the mean (SEM).

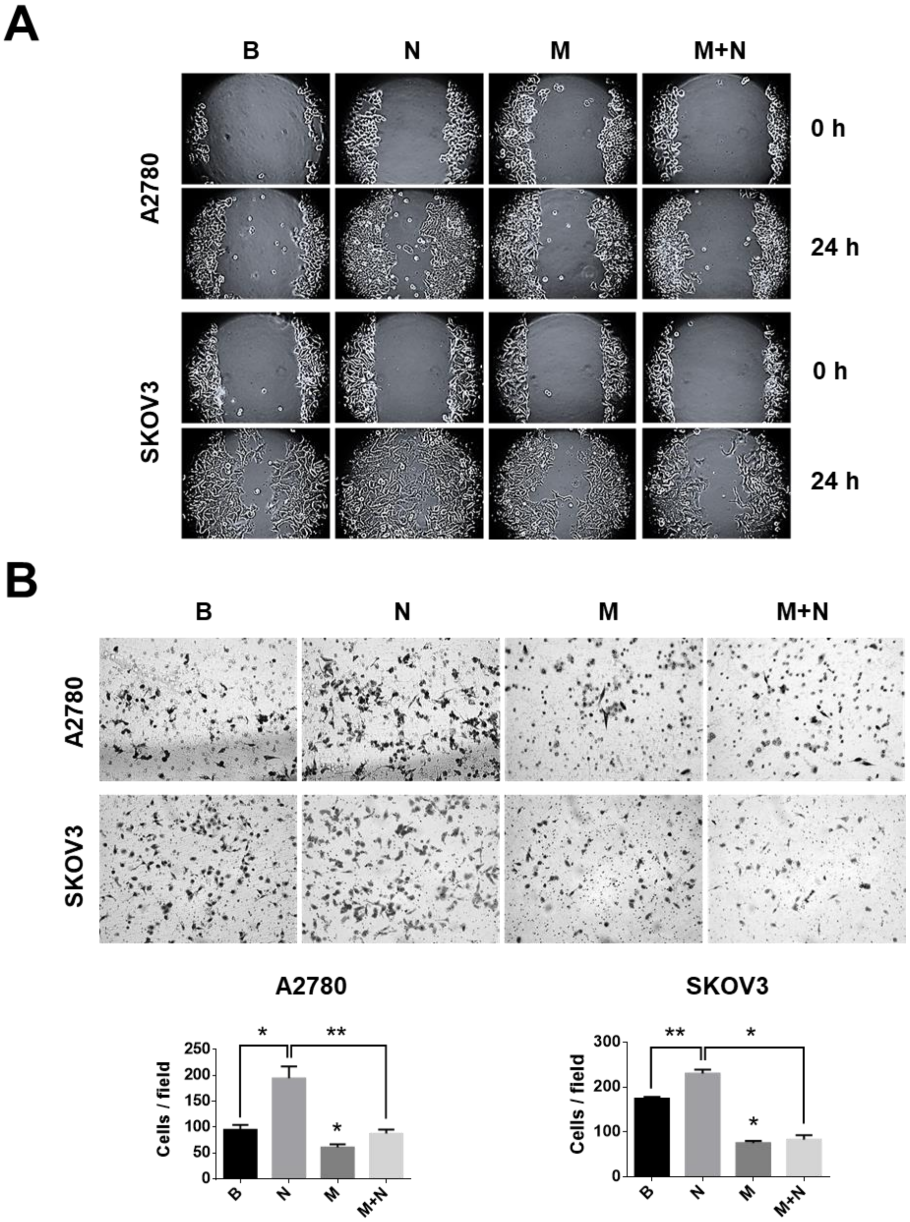

Supplementary Figure 9. Metformin prevents the increase in migration and invasion stimulated by NGF in EOC cells. (A) Wound healing assays performed with A2780 and SKOV3 cells stimulated with metformin (M, 10 mM) and NGF (N, 100 or 150 ng/ml). B: baseline condition (without stimuli). Ovarian cancer cells (500,000 per chamber) were serum-deprived for 24 h in 6-well plates. Wounds were introduced using a SPLScar^{TM} Scratcher (SPL Life Sciences, Korea). Immediately thereafter, EOC cells were stimulated with NGF (100 or 150 ng/mL for 24 h) and/or metformin and photographed at 0 and 24 h after the stimulation. **(B)** Invasion assays performed with A2780 and SKOV3 cells using the indicated conditions *=p<0.05 and **=p<0.01 with respect to the basal condition (Kruskal Wallis test and Dunn`s post-test). †=p<0.05 with respect to the basal conditions or as indicated using the Mann Whitney test. Results are expressed as the mean ± standard error of the mean (SEM).

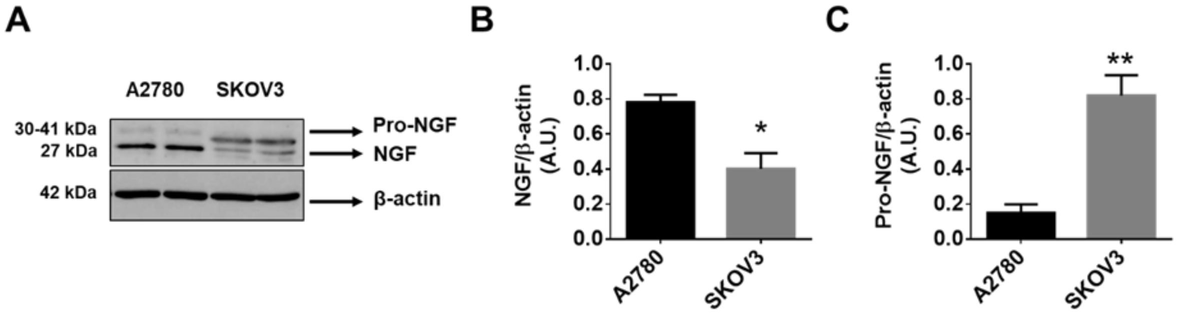

Supplementary Figure 10. **Immunodetection of NGF and Pro-NGF in EOC cells**. Protein extracts (50 ug) of A2780 and SKOV3 were used to determine NGF (A, B) and Pro-NGF (A, C) protein levels by western blotting with the primary rabbit polyclonal antibody ab6199 (Abcam, 1:500). N=4 (duplicate). *=p<0.05 and **=p<0.01 (Mann Whitney test).

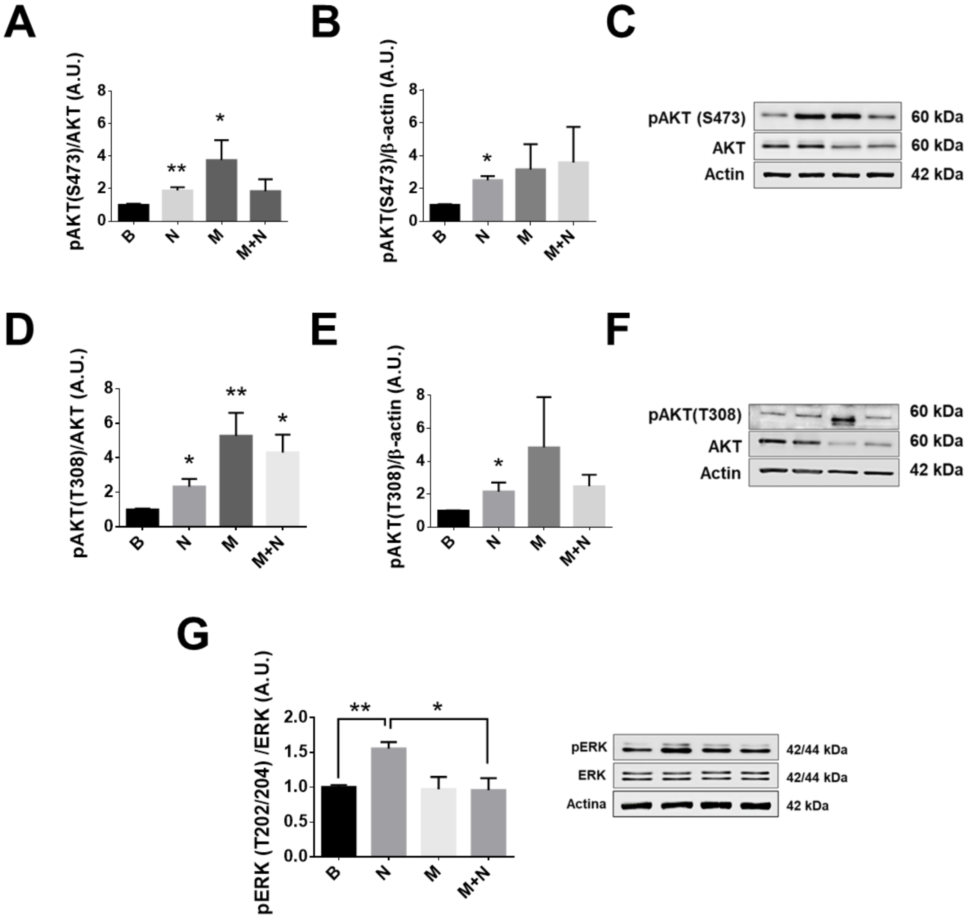

Supplementary Figure 11. Metformin prevents ERK activation by NGF in EOC cells. A2780 cells were treated with metformin (M, 10 mM, 48 h), NGF (N, 100 ng/mL, last 5 min) or no stimulus (B, basal condition). Then, cells were lysed and proteins (50 ug) were analysed by western blotting. **(A-C)** Representative western blot and the quantification of the bands by scanning densitometry to determine total AKT and phospho-AKT (S473) levels. **(D-F)** Representative western blot of total AKT and phospho-AKT (T308) and quantification by scanning densitometry. **(G)** Representative western blot and the quantification of bands by scanning densitometry for total ERK and phospho-ERK (T202/T204) in EOC cells. N=4 or more independent experiments. *=p<0.05, **=p<0.01 and ***=p<0.001 with respect to basal condition or as indicated (Kruskal Wallis test and Dunn`s post-test). Results are expressed as the mean ± standard error of the mean (SEM).
